# Designing minimal microbial strains of desired functionality using a genetic algorithm

**DOI:** 10.1186/s13015-015-0060-6

**Published:** 2015-12-21

**Authors:** Govind Nair, Christian Jungreuthmayer, Michael Hanscho, Jürgen Zanghellini

**Affiliations:** Department of Biotechnology, University of Natural Resources and Life Sciences, Vienna, Austria; Austrian Centre of Industrial Biotechnology, Vienna, Austria

**Keywords:** Systems biology, Metabolic networks, Elementary flux modes, Minimal cut sets, Strain optimization

## Abstract

**Background:**

The rational, in silico prediction of gene-knockouts to turn organisms into efficient cell factories is an essential and computationally challenging task in metabolic engineering. Elementary flux mode analysis in combination with constraint minimal cut sets is a particularly powerful method to identify optimal engineering targets, which will force an organism into the desired metabolic state. Given an engineering objective, it is theoretically possible, although computationally impractical, to find the best minimal intervention strategies.

**Results:**

We developed a genetic algorithm (GA-MCS) to quickly find many (near) optimal intervention strategies while overcoming the above mentioned computational burden. We tested our algorithm on *Escherichia coli * metabolic networks of three different sizes to find intervention strategies satisfying three different engineering objectives.

**Conclusions:**

We show that GA-MCS finds all practically relevant targets for any (non)-linear engineering objective. Our algorithm also found solutions comparable to previously published results. We show that for large networks optimal solutions are found within a fraction of the time used for a complete enumeration.

## Background

The availability of high amount of biological data has led to the reconstruction of genome-scale metabolic networks for many organisms [1–4] which can be analysed and probed using mathematical and computational methods [5, 6]. Prominent among these are constraint based modelling approaches which depend on the stoichiometry of the reactions. These include methods like flux balance analysis, FBA, [7] and elementary flux mode analysis, EFMA [8, 9]. The major difference between these approaches is that FBA seeks particular flux solutions whereas EFMA seeks to describe the entire flux space by enumerating all its elementary and balanced pathways which are called elementary flux modes, EFMs. Thus, the complete set of EFMs describes all possible cellular states. The disadvantage is that enumerating all the EFMs of a metabolic network is computationally very demanding as the number of EFMs explodes with network size [10]. However, the ability to enumerate EFMs has been steadily improving [11–14].

An important application of an EFMA is the prediction of gene knockouts to turn wild-type organisms into efficient minimal cell factories [15]. The design of efficient cell factories is based on the concept of networks of minimal functionality. These are derived from wildtype metabolic networks by keeping typically very few, specifically selected metabolic functions, e.g., EFMs with high yields of products of interest, while diminishing all other unwanted (wildtype) functionality by appropriately selected gene/reaction knockouts. These interventions channel the available carbon flux towards the product of interest. Based on EFMA the concept of constrained minimal cut sets, cMCS can be used to redirect cellular resources towards the product of interest [16]. cMCS are minimal (reaction) knock-out strategies, that disable unwanted EFMs (e.g., low product yield/growth) while the desired EFMs (e.g., high product yield) are preserved. In particular, cMCSs of minimal cardinality are important as these solutions minimize the experimental effort when knockouts are actually implemented in vivo. Several methods for the computation of cMCS based on a given EFM spectrum are known [16–18]. Alternatively, cMCS can also be calculated directly without first calculating EFMs [19–21]. However, in all these methods, explicit design criteria must be used (e.g. by providing boundaries for the desired minimal product yield). This is problematic in so far as a slight change in the design criteria might lead to large changes in the minimal cardinality of the cMCSs, i.e. the minimal number of required knockouts. For example, Trinh et al. [15] optimized *E. coli* for ethanol production with seven reaction knockouts. Jungreuthmayer et al. [22] on the other hand, were able to design a strain with identical key features and almost identical overall functionality, which required only five reaction knockouts.

If the EFMs are known it is theoretically possible but generally impractical to find all optimal partitions of EFMs and their corresponding cMCSs (of minimal cardinality). In a recent work Ruckerbauer et al. [23] approach this problem by first finding the smallest possible cMCS which contributes towards the engineering objective. Then cMCSs of higher cardinality are successively enumerated such that the engineering objective value is greater than or equal to that of the previous smaller cMCS. This circumvents the problem of large number of binning possibilities but will work, in a reasonable amount of time, only for small scale networks.

Here we present a novel approach which uses a genetic algorithm, GA to “evolve” near optimal solutions from starting sets of randomly partitioned modes. This results in minimal strains such that only that fraction of the total EFMs which contribute towards the design objective are active after deletion of the predicted cMCSs. This approach combines the simplicity of a GA with the power of EFMA and cMCS. The GA not only circumvents the manual partitioning of EFMs but also finds increasingly better solutions in a relatively short amount of time. This method can be used to satisfy not only traditional design objectives like product yield and growth but can also incorporate more complex design objectives like high growth-coupled product yield using minimal number of knockouts or even non-linear objectives.

## Preliminaries

### Elementary flux modes, EFMs

The material balances in a metabolic network with *m* internal metabolites and *r* reactions in steady state can be represented by

1 $$\begin{aligned} \mathbf N \cdot \mathbf v \, =\, 0. \end{aligned}$$

where $\mathbf N$ is the $\textit{m} \times \textit{r}$ stoichiometric matrix and $\mathbf v$ is a flux vector containing the fluxes through the network and $\mathbf v \in \mathbb {R}^{\textit{r}}$, i.e., $\mathbf v = (v_1,\ldots , v_r)^T$. The set of reactions can be partitioned based on thermodynamic constraints into sets of reversible and irreversible reactions. If *Irrev* is the index set of irreversible reactions,

2 $$\begin{aligned} v_{\textit{j}}\, \ge \, 0 \quad \forall \, \textit{j}\, \in \, \textit{Irrev}. \end{aligned}$$

The support of the flux vector $\mathbf v$ can be defined as $\text {supp}(\mathbf v )\, =\, \lbrace \textit{j} | v_{\textit{j}} \ne 0 \rbrace$, which is the set of reaction indices in $\mathbf v$ with non-zero flux values. An EFM, **e**, is a flux vector $\mathbf v \ne \mathbf 0$ which satisfies (1), (2) and a non-decomposability condition which states that, there is no non-trivial flux vector **w** satisfying (1), (2) and whose support is a proper subset of **e**, i.e., $\text {supp}(\mathbf w )\, \subset \, \text {supp}(\mathbf e )\,$. The non-decomposability condition means that the removal of any supporting reaction in an EFM will block a steady state flux through it. The set of all EFMs of a network completely describes the entire metabolic capabilities of the network. Every possible flux through the network can be expressed as a non-negative weighted combination of EFMs without cancellation. This means that if the flux through a reaction is 0, then all the contributing EFMs necessarily will have 0 flux through that reaction. For more information on EFMs, see [24].

We will use the following notation henceforth, $E = \text {supp}(\mathbf e )$. Let $\mathbf E =\lbrace E_1,\ldots , E_n \rbrace$ represent the full set of all *n* EFMs in support notation.

### Constrained minimal cutsets, cMCSs

Suppose there are certain network states which need to be suppressed. These states can be represented by a set of EFMs **T**, where $\mathbf T \subset \mathbf E$. The problem then becomes one of “killing” all the EFMs in **T**. This can be done by “knocking-out” a cutset *C* of reactions which will “hit” all of **T**. That is,

3 $$\begin{aligned} \forall \, \textit{T} \in \mathbf T , \; C \cap \textit{T} \ne \emptyset , \end{aligned}$$

*C* will be a minimal cut set, MCS, if there is no proper subset $B \subset C$ which satisfies (3) [25].

Suppose that in addition to network states which need to be suppressed, there are certain states which we need to preserve when knockouts are applied (e.g. biomass production and product formation). This can be done using the concept of cMCS [16]. The set of desired EFMs **D** corresponds to the network states to be preserved. Since in general it cannot be expected that an MCS will not hit any of **D**, we will say that we would like to have at least *k* EFMs untouched by an MCS where $\textit{k} \le \, \mid \mathbf D \mid$. Given an MCS *C*, let the set of EFMs $\mathbf D ^C$ represent $D \in \mathbf D$ which survive after applying *C*,

4 $$\begin{aligned} \mathbf D ^C = \lbrace D \in \mathbf D \mid C \cap D = \emptyset \rbrace . \end{aligned}$$

An MCS which satisfies (3) and the following constraint is a cMCS

5 $$\begin{aligned} \vert \mathbf D ^C \vert \ge \textit{k}. \end{aligned}$$

Thus an intervention problem

6 $$\begin{aligned} I = I(\mathbf T ,\mathbf D ,\textit{k}) \end{aligned}$$

is defined by a set of target EFMs **T** which need to be “killed” and a set of desired modes **D** of which at least *k* have to be “kept”. Several methods to solve (6) are available [16–18]. Note that $\mathbf {D} \cup \mathbf {T}$ does not necessarily unite to the full set of EFMs since there could be EFMs which we do not want to either kill or keep but instead have a “don’t care” status. However, we do not need to specify such an association since we will not operate on these EFMs. We will operate only on the EFMs we are interested in ($\mathbf {D}$ and $\mathbf {T}$) and do not bother with what happens to the EFMs with “don’t care” status because by definition it wouldn’t matter to us if these EFMs survive or are killed.

In the following we describe a GA to solve the intervention problem (6). For simplicity our implementation partitions the complete set of EFMs into $\mathbf {D}$ and $\mathbf {T}$ and does not make use of the “don’t care” option.

## Methods

### The EFM kill/keep problem

Equation (6) allows to search for cMCS which keep certain EFMs and kill others. However, it is not intuitive which EFMs to keep and which to kill in order to minimize the cardinality of the cMCSs. Thus the question arises: What is the best partitioning of EFMs in order to reach a specific engineering objective? Even in a modest sized network, the possible combination of EFMs to keep or kill is very large. For example, in a small scale network with 5000 EFMs, the number of possible kill/keep combinations is $2^{5000}$. It is practically impossible to explore all points in such a large solution space. Therefore, it makes sense to utilize a program that finds the best set of EFMs to keep, and the corresponding cMCSs which will achieve this for a given an engineering objective [23]. We do this using a GA, the working of which is described below.

### The genetic algorithm, GA

GAs are heuristics inspired by the theory of evolution, generally used when the extreme of the function cannot be analytically established or when it is impractical to search the whole solution space. GAs work on problems by encoding possible solutions into a population of individuals. These individuals are chromosome like data structures which are iteratively refined to “evolve” better solutions by applying strategies inspired by Darwinian evolution [26–29]. In our implementation each individual represents an intervention problem (6).

Given a population size *p*, we randomly generate individuals $S_i=\{s_i^1,\ldots ,s_i^n\},\ 1\le i\le p$, where each element $s_i^j$ of $S_i$ indicates if the EFM $E_j$ is present ($s_i^j = 1$) in the individual $S_i$ or not ($s_i^j = 0$). Thus each individual $S_i$ codes an intervention problem (6) with

7 $$\begin{aligned} \begin{array}{l} I_i = I_i[\mathbf {T}(S_i),\mathbf {D}(S_i),k(S_i)],\\ \quad \text {with } \mathbf {T}(S_i) = \{E_j \vert s_i^j = 0\},\\ \quad \quad \; \mathbf {D}(S_i) = \{E_j \vert s_i^j = 1\},\\ \quad \quad \; k(S_i) = w_k \vert \mathbf {D}(S_i) \vert \end{array} \quad 1\le i\le p, 1\le j\le n \end{aligned}$$

where $w_k \in [0,1]$ is a freely adjustable GA parameter. $s_i^j$-values are assigned randomly but we provided for the possibility to pre-process EFMs such that EFMs with desirable characteristics have a higher chance of being 1. For example, suppose a cell is described by the following set of EFMs $\lbrace E_1,\ldots , E_7 \rbrace$, where only $E_1$, $E_3$ and $E_7$ support product formation. If we want to optimize for product formation, we clearly do not want to keep the non-producer. So we choose $s_i^j$ such that undesirable states never get selected. In our example possible randomly selected individuals could look like $S_1 = \lbrace 1,0,1,0,0,0,1 \rbrace$, $S_2=\lbrace 1,0,1,0,0,0,0 \rbrace$, etc. while $\lbrace 1,1,1,0,0,0,1 \rbrace$ would not be generated because it includes $E_2$ which we want to eliminate. This leads to a significant reduction in the search space. Finally, for each individual $S_i$, cMCS are calculated using the MHScalculator [30].

GAs aim to proceed towards better solutions by evaluating each individual $S_i$ against a fitness function *F* and selecting the top-performers for procreation. The fitness function reflects the design objective since those are the traits we want to improve. In our implementation individuals are selected for mating using a fitness proportionate selection [31]. In addition, we use the concept of “elitism” where a pre-specified percentage of top-performers will propagate into the next generation without any modification as shown in Fig. 2c. This guarantees that the population’s maximum fitness does not decrease. We use crossover, mutation [26, 27], and random selection based on previous information about surviving EFMs to produce a new generation of individuals. These mechanisms are explained below.

#### Crossover

We take two parent individuals, $S_1$ and $S_2$, and randomly exchange their elements to create two new offspring $S_3$ and $S_4$. We implemented the following three standard types of crossovers. For 1point crossover, generate a random integer $r_c, \, 1 \le r_c<n$ for each pair of parents, then the offspring of crossover are $S_3 = \lbrace s_1^1, ... s_1^{r_c}, s_2^{r_c + 1}, ... s_2^n \rbrace$ and $S_4 = \lbrace s_2^1, ... s_2^{r_c}, s_1^{r_c + 1}, ... s_1^n \rbrace$, (see Fig. 2a). In 2point crossover, two random integers $r_{c1}, r_{c2}, \; 1 \le r_{c1}<r_{c2}<n$ are generated for each pair of parents. The offspring in this scenario are $S_3 = \lbrace s_1^1,... s_1^{r_{c1}}, s_2^{r_{c1} +1},... s_2^{r_{c2}}, s_1^{r_{c2} + 1},... s_1^{n} \rbrace$ and $S_4 = \lbrace s_2^1,... s_2^{r_{c1}}, s_1^{r_{c1} +1},... s_1^{r_{c2}}, s_2^{r_{c2} + 1},... s_2^{n} \rbrace$. In uniform crossover, for each EFM a random number $0 \le r_u^j < 1$ is generated and the offspring are $S_3 = \lbrace s_1^j \quad \text {if} \; r_u^j < 0.5 \; \text {else} \; s_2^j \rbrace$ and $S_4 = \lbrace s_2^j \quad \text {if} \; r_u^j < 0.5 \; \text {else} \; s_1^j \rbrace$.

#### Mutation

Given an individual $S_1$ and a random integer *r*, $1 \le r < n$, the mutated individual is $S_{2} = \lbrace s_1^i \quad \text {if} \; i \ne r, \; \text {else} \; 1 - s_1^i \rbrace$. The absolute number of such random integers generated for each individual is given by $\rho r_m$, where $r_m$ is a freely adjustable GA parameter, the mutation rate, $0 \le r_m < 1$ and $\rho$ the maximum number of EFMs with desirable characteristics, $\rho \le n$ (see Fig. 2a).

#### Pattern-based individual generation

In addition to mutation and crossover we create new individuals based on the fittest patterns. For each individual *S*, whose corresponding intervention problem has solution(/s), we generate a “design pattern”, which contains only the surviving EFMs,

8 $$\begin{aligned} P = \lbrace p^j \mid p^j = 1 \quad \text { if } E_j \in \mathbf D ^C\text { else }p^j = 0 \rbrace . \end{aligned}$$

Given a binary individual $S \, =$ 1010001, if only EFM 3 and 7 survive the intervention, the resulting pattern will be 0010001. Thus a pattern is a specific strain design for an intervention problem. A solvable intervention problem typically produces more than one solution. Therefore, one individual will usually have more than one pattern associated with it. Since the fitness depends on the surviving EFMs, each pattern will have its own fitness value. Thus one individual may be associated with more than one fitness value. Here, the fitness of an individual *S* is defined as the fitness of the fittest pattern *P*.

To create the new individuals, we start by weighting each EFM proportional to the number of times the EFM survived in all previous patterns. Let ${\varvec{P}}_t$ represent the entire set of patterns found until a given generation *t*. The weight $w^i_t$ for an EFM $E_i$ is calculated by

9 $$\begin{aligned} w^i_t = \sum _{j = 1}^{\vert {\varvec{P}}_t \vert } ({\varvec{P}}_t)^i_j. \end{aligned}$$

Next we generate a set of desired candidate EFMs by randomly selecting a random number of EFMs with non-vanishing $w^i_t$. Out of these desired candidate EFMs new individuals were composed by including those candidate EFMs for which a randomly selected number $r_i$ was not larger than the weight of the corresponding candidate EFM, $0 \le r_i < \max w_t$ and $\max w_t$ is the maximum of all such weights (see Fig. 2b),

10 $$\begin{aligned} S_{\text {new}} = \lbrace s_{new}^i | \quad \text { if } w^i_t \ge r_i, \, s_{new}^i = 1 \text { else } s_{new}^i = 0 \rbrace . \end{aligned}$$

The number of individuals generated by this method can be controlled by the GA parameter ‘new_S’, Table 1. It is a way to consider all good solutions obtained so far and ensures that more EFMs with desirable properties find their way into the set of desired EFMs. This helps the GA to reach the optimum faster.

**Table 1:** The GA parameters

No	GA parameter	Description
1	*t*	This parameter is used to specify the number of generations for which the GA will run
2	*p*	This parameter is used to specify the number of individuals *S* present in one generation of the GA
3	$r_m$	This parameter is used to set the mutation rate which specifies the number of bits in an individual *S* that will be flipped from 0 to 1 or vice versa
4	cross	This parameter is used to select among the three types of crossover operations possible here: 1point, 2point and uniform
5	elit	This parameter is used to specify the fraction of the number of total individuals from the previous generation which will be retained in the subsequent generation
6	new_S	This parameter specifies the number of new individuals which will be generated in each generation, based upon information from previous generations
7	t_stop	This parameter is used to set the maximum number of generations after which the GA terminates if the maximum fitness remains unchanging
8	min_1s	This parameter specifies the fraction of maximum number of possible good modes which must be present in the initial population
8	$w_k$	This parameter is used by the MHSCalculator to specify the minimum number of EFMs which have to survive in given a set of desired modes **D** (provided as fraction of the number of EFMs in **D**)
9	threads	This parameter specifies the maximum number of threads to be used by the program

These parameters are used to control the running of the GA and also to get more specific results

**Fig. 1:**
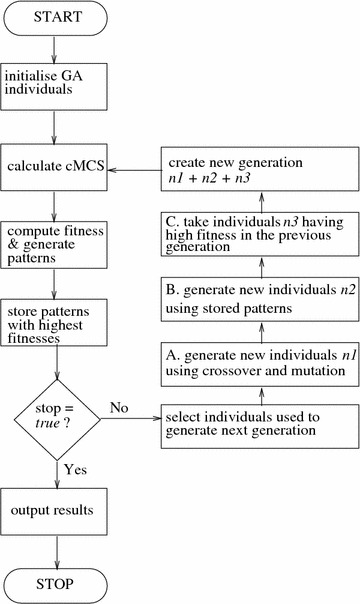
Flowchart of the GA. The GA stops when a stopping condition is met, which here is if the number of generations reaches a pre-specified maximum or if the maximum fitness remains unchanged for a pre-specified number of generations (Table 1)

**Fig. 2:**
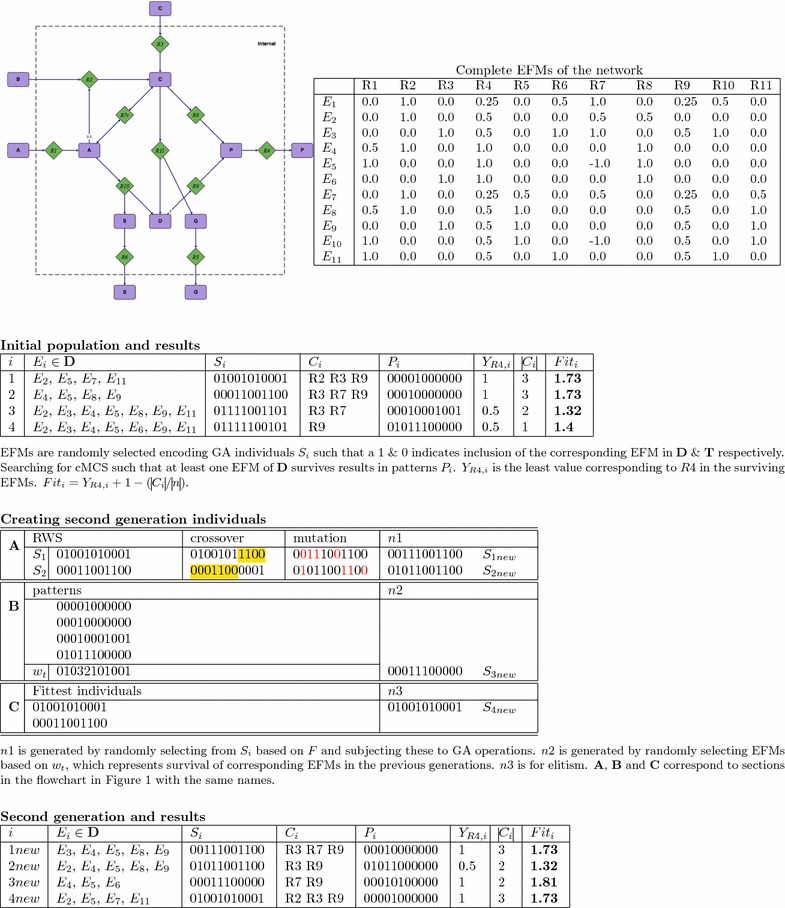
GA example. Running the GA on the given toy network of 11 EFMs with the aim of maximizing production of P. The initial individuals $S_i$ and the effect of applying the mutation, crossover and elitism operators to generate new individuals are shown. Here the GA finds the best solution with a fitness of 1.81 and yield ($Y_{R4}$) of 1 in the second generation

The GA stops after reaching a pre-specified number of generations or when the maximum fitness doesn’t improve for a given number of generations, outputting all MCSs of minimal cardinality associated with each desired pattern. The schematic of the GA implemented and used here is shown in Fig. 1 along with a small illustratory example in Fig. 2.

### Implementation

The GA was implemented in Perl http://www.perl.org/. cMCSs were calculated with MHScalculator which is an open source C-program that is freely available [30]. EFMs were calculated using the *regEfmtool* [13]. All runs were performed on a machine with the following specifications—2 CPUs, 12 cores, Intel Xeon X5650 2.67 GHz and an Ubuntu 14.04 LTS operating system, allowing the used programs to utilise 10 threads in parallel. Caching in form of look-up tables is employed to store previously obtained MCS, patterns and corresponding fitnesses, to avoid repetition of calculation. We also use *tmpfs*, a temporary file storage created on the RAM, for faster i/o on intermediate files. A general description of the parameters used for controlling the GA are shown in Table 1. Specific parameter values for the individual runs are shown in Table 2.

**Table 2:** GA parameters for different runs

GA parameter	M1 ethanol	M1 efficiency	M1 complex	M2 ethanol	M2 efficiency	M2 complex	M3 ethanol	M3 efficiency	M3 complex
$w_{1}$	1	0	1	1	0	1	2	0	1
$w_{2}$	0	50	50	0	50	50	0	10	50
$w_{3}$	1	1	1	1	1	1	1	1	1
$w_{4}$	1	1	1	1	1	1	1	1	1
*t*	100	100	100	100	100	100	100	100	100
*p*	50	50	50	50	50	50	50	50	50
$r_m$	0.00025	0.00025	0.00025	0.00025	0.00025	0.00025	0.000025	0.000025	0.000025
cross	1point	1point	1point	1point	1point	1point	1point	1point	1point
elit	0.025	0.025	0.025	0.025	0.025	0.025	0.025	0.025	0.025
$w_k$	0.03	0.017	0.04	0.025	0.01	0.03	0.01	0.0075	0.03
new_S	0.1	0.1	0.1	0.1	0.1	0.1	0.1	0.1	0.1
t_stop	15	15	15	15	15	15	15	15	15
min_1s	0.9	0.9	0.9	0.9	0.9	0.9	0.9	0.9	0.9
threads	10	10	10	10	10	10	10	10	10

Parameters used in the various runs

### Validation

We ran the GA on an *E. coli* core model, M3, [15] and two smaller models, M1 and M2, which were derived from the parent model, M3, by removing several reactions. M3 describes the central carbon metabolism of *E. coli* including the uptake and utilization of several hexose and pentose sugars. Compared to M3, M2 is restricted to model only glucose utilization (all other carbon uptake relations were removed). Finally, M1, the smallest model of the three, describes glucose utilization under anaerobic conditions. The main topological properties of the three models are summarized in Table 3.

**Table 3:** Features of models used

Model	M1	M2	M3
Model source	[15]	[15]	[15]
Growth conditions	Anaerobic, glucose + minimal media	Aerobic, glucose + minimal media	Aerobic, xylose, arabinose, glucose, galactose and mannose + minimal media
No: reactions	59	60	71
No: metabolites	47	49	68
Total no: EFMs	5010	38001	429275
*F*_1_	1.6170	1.6103	2.2770
$\max Y_{Etoh}$	0.6667	0.6667	0.6667
MCS cardinality	3	4	4
Number of MCSs	22	82	76
Number of EFMs	14	28	62
*F*_2_	7.7860	8.5283	2.3169
$\max \eta _{Etoh}$	0.1390	0.1542	0.1542
MCS cardinality	10	13	16
Number of MCSs	240	240	2880
Number of EFMs	4	2	6

Features of the networks on which the GA was tested. The maximum possible values for ethanol yield, $Y_{Etoh}$ and efficiency, $\eta _{Etoh}$ are presented. The minimal cardinality of MCSs which will force the network into these optimal values are also shown along with the total number of such MCSs and the number of EFMs which will survive after application of these MCSs. The corresponding fitness values, $F_i$ have been obtained using the fitness functions presented in Table 4

## Results

Our aim is to design optimised *E. coli* strains for ethanol production. The optimization objectives considered in this study were ethanol yield ($Y_{Etoh}$), substrate specific productivity which is the product of normalised specific ethanol production and normalised biomass production [32] also called “efficiency” ($\eta _{Etoh} = Y_{Etoh} \times Y_{Biomass}$), and an objective which considers both the yield and efficiency together. In all objectives, we favour solutions with low cardinalities (for details see Table 4).

**Table 4:** Fitness functions used

*i*	Design objective	Fitness function $F_i$
1	Ethanol production with minimal MCS size	$w_{1} \min Y_{Etoh} + w_{3} (1 - \vert C \vert /n)$
2	Substrate specific productivity with minimal MCS size	$w_{2} \min \eta _{Etoh} + w_{3} (1 - \vert C \vert / n)$
3	Growth coupled product yield with minimal MCS size and maximum number of surviving modes	$w_{1} \min Y_{Etoh} \times w_{2} \max \eta _{Etoh} + w_{3} (1 - \vert C \vert / n) + w_{4} \vert \mathbf D ^C \vert / \vert \mathbf E \vert$

Fitness functions used, where, $w_{1}$, $w_{2}$, $w_{3}$ and $w_{4}$ are weights associated with ethanol yield ($Y_{Etoh}$), ethanol efficiency ($\eta _{Etoh}$), MCS cardinality ($\vert C \vert$) and number of surviving modes ($\vert \mathbf D ^C \vert$) respectively. These weights are used primarily to ensure desired contribution of the different variables towards the fitness function. They can also be used to give higher preference to a particular variable. *C* is the MCS, *n* the total number of reactions and **E** the set of all EFMs in a network. All fitness functions were maximised

### Benchmarking

We tested the performance of the GA against the automatic partitioning method, APM developed by Ruckerbauer et al. [23] using the models M1, M2 and M3. The APM was selected for comparison, as for any given, linear engineering objective APM enumerates all optimal knockout strategies without requiring any manual interference. We tested for maximum efficiency and ethanol production using the fitness function $F_1$ and $F_2$, respectively as given in Table 4. For the three models used we listed the main characteristics of the optimal solutions with respect to the fitness functions in Table 3. All simulations were run five times. In the following we reported averages over these five runs, unless otherwise stated.

#### Maximizing for efficiency

We used the fitness function $F_2$ (Table 4) with the parameters shown in Table 2 to optimize for efficiency. The GA was terminated when the fitness function remained unchanged for 15 generations.

**Fig. 3:**
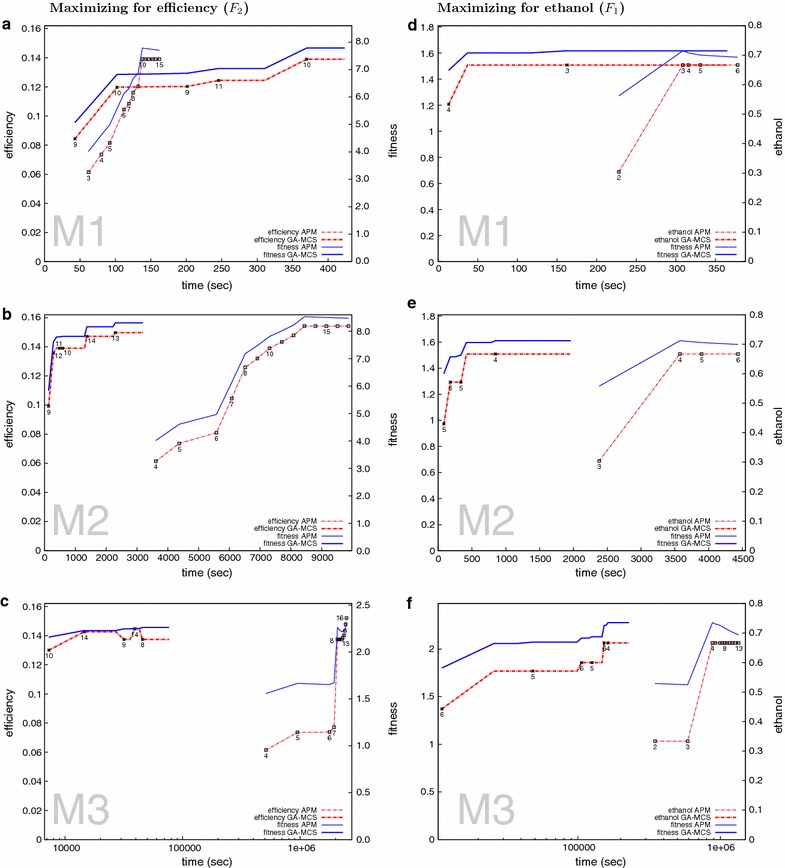
GA performance comparison. Comparing the performance of GA-MCS against APM [23] using a single representative run for each model. The best solution in each generation was used to represent the performance of the GA. The *numbers* under the *lines* represent the cardinality of MCS corresponding to the objective value plotted. **a**, **b** and **c** represent maximization for efficiency and **d**, **e** and **f** represent maximization for ethanol using the corresponding fitness functions in Table 4. M1, M2 and M3 indicates the associated models. The time axes in **c** and **f** is in logarithmic scale. Note that for the same objective with a lesser cMCS cardinality, the fitness will be higher

The GA found all optimal solutions for the small model M1 (see Fig. 3a). In the bigger models M2 and M3 the GA did not find the best solutions but got within 3 and 1.2  % of the maximum fitness, respectively.

In M2 and within the selected runtime, the GA mostly found near optimal solutions (see Fig. 3b), and rarely converged to the optimal solution. In the case of M3 the GA got stuck in a local optimum (see Fig. 3c).

While the GA does not necessarily identify the absolute best solutions, it generally finds near-optimal solutions extremely quickly. In M2 and M3 near-optimal solutions are found in about 25 and 2.5 % of the time taken by the APM, respectively (see Fig. 3). Only in the small-scale model M1, which is easy to enumerated fully, the GA is slower than the APM.

**Fig. 4:**
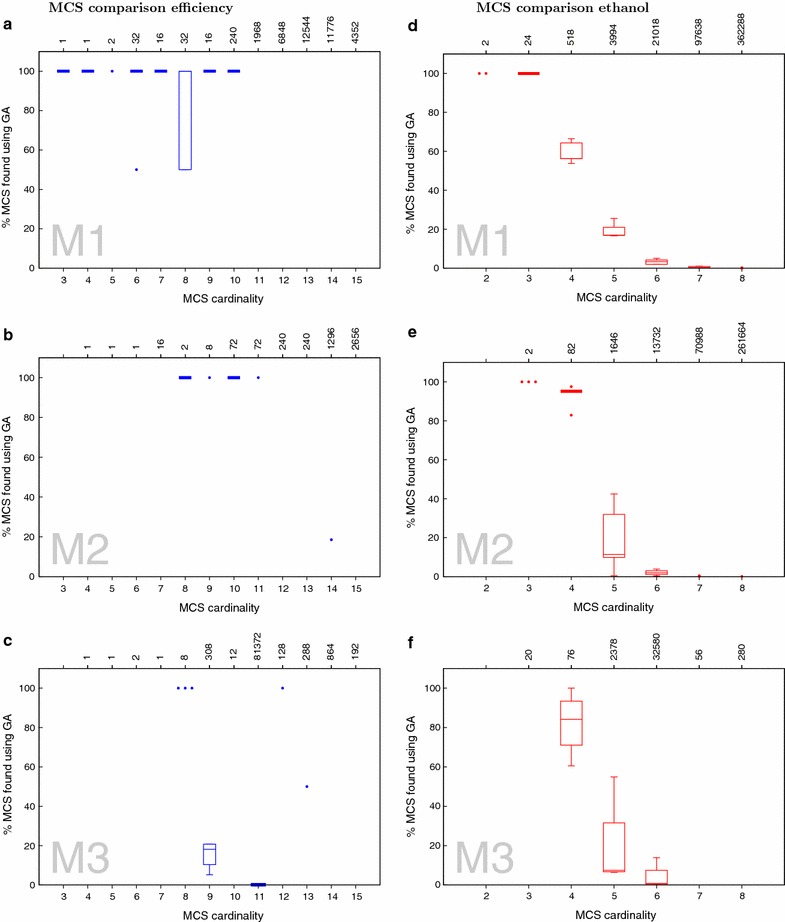
Number of solutions retrieved by the GA. *Boxplots* representing the number of matches between MCS retrieved using GA-MCS and APM broken down by cutset cardinality across five runs. *Boxes* have been drawn around the first and third quartile values, with the median being represented by the *horizontal line* within the* box*. *Points* represent outliers or data with three or lesser number of points. The *numbers* shown at the *top of each plot *indicate the total number of MCSs of the given cardinality as found by APM. **a**, **b**, **c** represent results for efficiency maximization and **d**, **e**, **f** for ethanol maximization for the indicated models M1, M2 and M3

Comparing the MCSs obtained with the GA to the ones obtained with the APM, as shown in Fig. 4a–c, reveals that our algorithm retrieves 100 % of all low cardinality MCS. The number drops with increasing MCS’ cardinality. This behavior is expected as our fitness functions favors low cardinality solutions. Thus it is very unlikely that the GA will identify many high cardinality solutions. In fact, this explains the non-monotonic behavior of the line of maximum efficiency in Fig. 3c. Because the fitness function $F_2$ allows for a trade off between cardinality and maximum efficiency, the efficiency might decrease. Yet the fitness function still increases.

#### Maximizing for ethanol production

We used the fitness function $F_1$ (Table 4) with the parameters shown in Table 2 to maximise for ethanol yield. The GA was terminated when the fitness function remained unchanged for 15 generations.

Unlike the previous case, here our algorithm found all optimal solutions for all models. Also, we were faster than the APM in reaching the optimum for all models (see Fig. 3d–f).

Again, like in the case of maximising for efficiency, the GA retrieves 100 % of lower cardinality MCSs (Fig. 4d–f), and not many of the higher cardinality solutions, when compared to the solutions obtained using APM. This is a result of the fitness function, $F_1$, which favors towards lower cardinality MCSs. The effect of this can be observed in Fig. 3d, e where the GA first finds higher cardinality solutions for the optimal ethanol yield and settles down to the lowest possible cardinality in subsequent generations.

### Optimizing for a complex design

Although maximising for ethanol yield and efficiency, produces sub-optimal to optimal designs, these designs may not be the best to implement in vivo. For example, the EFMs which result in the maximum ethanol yield do not support growth. However, two of these EFMs provide maintenance energy. On the other hand, designs with maximum efficiency do not include maximum ethanol producing EFMs. It would be preferable to have a design which combines these features. We used the fitness function $F_3$ (Table 4) with the parameters shown in Table 2 to find optimal designs.

**Fig. 5:**
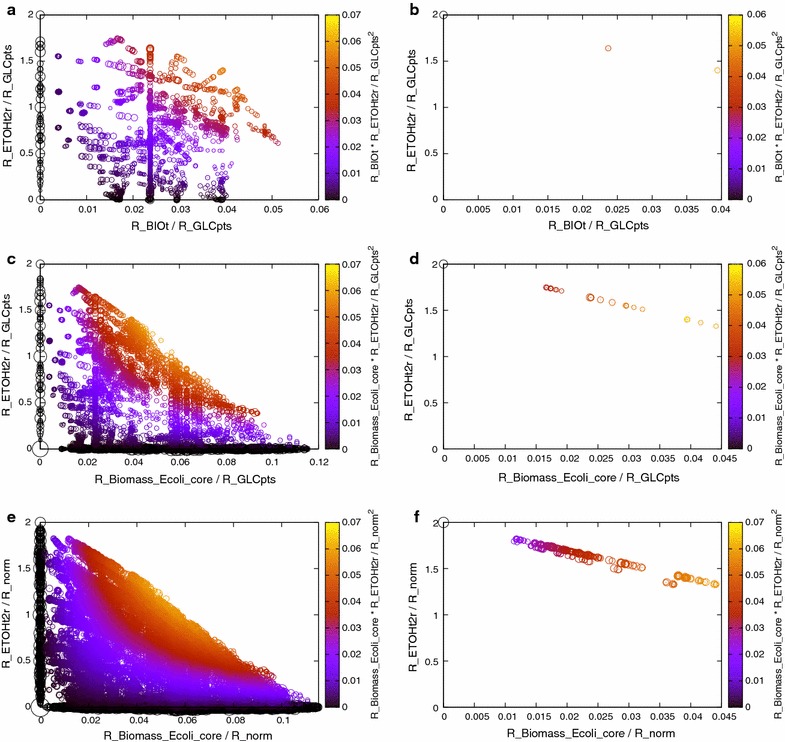
Complex designs optimized by the GA. **a**, **c** and **e** show the complete set of EFMs of the M1, M2 and M3 models respectively and **b**, **d** and **f** represent corresponding solutions obtained using the GA which were obtained in 22, 28 min and 8 h, 48 min respectively. EFMs are represented as a function of ethanol and biomass production. *Each circle* represents a set of EFMs with the same yield and efficiency. The *diameter of the circle* reflects the number of EFMs represented. The *colour* of the EFMs indicates their efficiency as specified by the index on the *right hand side* of each graph. R_ETOHt2r, R_BIOt and R_GLCpts represent the ethanol secretion, biomass and glucose uptake reactions in the model. In **b**, the cutset corresponding to the solution is ({R_G6PDH2r R_FRD7 R_LDH_D R_ACt2r R_SUCCt3}), in **d**, the modes represented are the ones which survive after applying the cutset ({R_GND R_FUM R_ACt2r R_D_LACt2 R_SUCCt3}) and in **f** the cutset corresponding to the solution is ({R_GND R_SUCOAS R_MALS R_ACt2r R_D_LACt2}). In **e** and **f** R_norm = R_GLCpts + R_MAN1 + R_TRA8 + R_TRA9 + R_TRA10

A similar problem was looked at in [23] where the authors optimised M1 for efficiency while ensuring that at least one of the maximal ethanol producing EFMs survive in the final design. Their design included the most efficient ethanol producing EFMs as well as EFMs with maximum ethanol yield, achieved with an MCS cardinality of 6. A similar design was used by Trinh et al. [15] using 7 reaction knockouts. Our algorithm produces designs of similar functionality with MCSs of cardinality 5, Fig. 5b. Similar results were obtained for M2, and M3 as shown in Fig. 5d and f respectively, both with MCS cardinalities of 5. Also, our algorithm was very quick in finding these designs, taking a few minutes for M1 and M2 and a few hours for M3.

## Conclusion

We have presented a method for the design of minimal microbial strains of desired functionality. The designs are minimal in the sense that only a few of the total number of pathways (EFMs) are active after deletion of the predicted cMCSs. Our GA uses the MHScalculator [30] to find cMCSs for a given set of desired and target EFMs. However, the optimal selection of such sets is non-intuitive. Hence, the aim was finding the best possible set of pathways which maximise a given engineering objective.

Another GA, called the OptGene method has been previously reported which finds reaction cuts to achieve a design objective [33]. This algorithm works by testing different combinations of reaction knockouts. In contrast, we test partitions of EFMs. Thus our search space is by orders of magnitude larger than theirs. OptGene finds many solutions too, but it cannot be guaranteed that these are minimal. Also, the knockout cardinalities are restricted to 1–10. Our approach is based on the concept of EFMs which enumerate all possible network states. OptGene however uses methods like FBA, MOMA [34], etc. to calculate the fitness which, unlike EFMs do not account for alternative pathways. Although methods which use FBA and MOMA predict optimal solutions, there is no guarantee that the predicted optimum will be achieved. In a similar vein, the method presented here has advantages over other methods which use a biased biological objective like OptKnock [35], RobustKnock [36] and tilting of the objective function [32].

Boghigian et al. [37] also use a GA and EFMs to design strains with higher product yields. Their approach however differs from the method presented in this paper in a few major ways. First, the aim of the GA presented in [37] is to only improve product yields without considering the minimality of the knockouts. Hence, in contrast to us their predicted knockouts are not guaranteed to be minimal. Second, the basic problems considered by both methods are different, although the final aim is the same, namely strain improvement. Boghigian et al. look for reaction knockouts which will improve product yields. Our GA not only maximizes the product yield but also simultaneously searches for optimal partitions in the set of EFMs. Finally, we deal with networks where the number of EFMs are one order of magnitude larger than that used in [37].

Tools which use EFMs to find intervention strategies include the MHScalculator [17, 30] and a tool to calculate cMCSs as part of the *CellNetAnalyzer*, a MATLAB package providing comprehensive structural and functional analysis of biochemical networks [38]. These methods use EFMs and hence consider the entire metabolic landscape of the organism. The limitation of these methods is that the EFMs which must survive or be killed by an intervention have to be manually partitioned.

A recent method (APM [23]) overcomes this issue by calculating all partitions of EFMs for MCSs of increasing cardinality such that the objective is higher than that corresponding to the previous smaller MCS size. This is an exhaustive and exact method for finding intervention strategies in metabolic networks. However, this method is impractical for large networks given current computational capabilities. Although the GA is not faster than the APM at very small network sizes like M1, its comparative performance improves with increasing network sizes, Fig. 3. Also, when optimizing for efficiency, the GA does not reach the global optimum when APM does, Fig. 3b, c. Note that however, an exact comparison to APM is not possible since APM tries to find all MCS whereas the GA tries to find the best cut set for a particular objective. Our method also incorporates the freedom to encode complex design criteria, which is not possible with the APM. Also, since the APM is based on linear programming, it is limited to linear objective functions whereas we can implement non-linear objective functions as well.

An important new approach initially proposed by Ballerstein et al. [19] with further improvements in [20, 21] is able to directly find MCSs without first needing to calculate the EFMs by using the concept of hypergraph dualisation. This gets rid of the problem of explosion in the number of EFMs with increasing network sizes, allowing for prediction of intervention strategies in genome-scale metabolic networks. However, these methods have to specify design criteria like minimal product yield [20]. This is a limitation in that slight changes in the value of the specified design criteria may lead to different MCSs. In contrast, our algorithm tries to automatically find the best design criteria.

The GA implemented here is able to predict numerous good solutions to problems of product maximization which are comparable to experimentally verified designs [15]. One advantage of this method is the short time taken while dealing with bigger systems. The biggest advantage though is the flexibility in the selection of the design criteria using the fitness function. The fitness function can be arbitrarily complex to accurately reflect the design criteria. Here it has allowed us to produce good designs without knowing the specific properties of EFMs which need to survive.

Since our approach mainly relies on a GA, it may be affected by inherent limitations of GAs, including the possibility of getting stuck at a local optimum. This may be overcome by employing multiple runs or changing the GA parameters. Note that we have considered reaction knockouts here but this can be easily translated into gene knockouts using gene-reaction associations.

Finally, we provide a brief description of the parameter values used. The mutation rate was set such that only two to four positions in an individual are affected, an increase in this number resulted in the GA not producing any good solutions. Decreasing this number resulted in a slower rate of improvement in fitness (data not shown). It is also possible to completely turn off mutation by setting $r_m$ to 0. In any case the performance of the GA can be improved with pattern-based individual generation rather than relying solely on mutation and crossover. The number of such individuals can be adjusted with the ‘new_S’ parameter. However, too high ‘new_S’ values led to a comparatively worse GA performance (data not shown). The parameter $w_k$ specifies the minimum number of EFMs which should survive an intervention. The lesser this value, the higher the probability of finding better solutions—because typically, optimal solutions have very few surviving EFMs, Table 3. However, small $w_k$ also produces more solutions which in turn takes more time for pattern and fitness calculations. In order to reach the optimum with as few solutions as possible, we found that in general, $w_k$ can be large for small models (e.g., M1) and must decrease for growing models (e.g., M2 and M3) (for exact values see Table 4). ‘min_1s’ determines the minimum number of possible good EFMs that will end up in the set of desired EFMs **D** in the initial population. Because the EFMs are randomly selected to be in **D**, not all individuals will generate viable solutions. Also, it is important that the union of **D**s in the whole population nearly covers the set of good EFMs. The EFMs which are not covered must otherwise rely on mutation to be transferred from **T** to **D**. The probability of this happening decreases with increasing individual size. Hence, ‘min_1s’ was set to a high value of 0.9 for all of the runs. A future direction of this work would be to study the effect of these parameters in detail. This will help get rid of the empirical setting of parameters in our GA and allow for the implementation of a protocol to automatically determine these values during the running of the GA.

In summary, our algorithm is able to quickly find (near) optimal intervention strategies satisfying non-linear engineering objectives in large metabolic networks. However, EFMs are still necessary for our method which is a significant bottleneck when it comes to genome-scale networks. We expect that combining the dual method [19–21], which will allow for the calculation of cMCS directly from the stoichiometric matrix, with a GA will overcome this hurdle.
